# The deformities of acute diaphyseal clavicular fractures: a three-dimensional analysis

**DOI:** 10.1186/s12938-023-01112-z

**Published:** 2023-05-10

**Authors:** Yi-Hsuan Chao, Ying-Chao Chou, Chun-Li Lin

**Affiliations:** 1 Department of Biomedical Engineering, National Yang Ming Chiao Tung University, No. 155, Sec. 2, Linong Street, Taipei, 112 Taiwan; 2 Department of Orthopaedic Surgery, Taipei City Hospital, No. 10, Sec. 4, Ren’ai Rd., Da’an Dist., Taipei, 106 Taiwan; 3 Innovation & Translation Center of Medical Device, National Yang Ming Chiao Tung University, No. 155, Sec. 2, Linong Street, Taipei, 112 Taiwan; 4 Department of Orthopedics, Chang Gung Memorial Hospital, No. 5, Fuxing St., Guishan Dist., Taoyuan, 333 Taiwan

**Keywords:** Clavicle, Fracture, Diaphyseal clavicular fracture, Shortening, Computed tomography, 3D

## Abstract

**Background:**

Although minimally invasive surgeries have gained popularity in many orthopaedic fields, minimally invasive approaches for diaphyseal clavicular fracture have not been widely performed, which is attributed to difficulties in performing a closed reduction of fracture deformities of a curved bone in a three-dimensional space. The goal of this study was to investigate the radiographic parameters of fracture deformities in a three-dimensional space and to identify the risk factors for deformities.

**Methods:**

The computed tomography images of 100 patients who sustained a clavicle fracture were included. Five parameters were used to analyze the deformities: change in clavicle length, fracture displacement, and fragment rotation around the X, Y, Z axes. The change in length was assessed using the length of the endpoint line. The displacement was assessed using the distance between the fracture midpoints. The rotation deformities were assessed using the Euler angles. The correlation between the parameters was evaluated with the Pearson correlation coefficient. The risk factors were evaluated using univariable analysis and multiple regression analysis.

**Results:**

The average change in length was − 5.3 ± 8.3 mm. The displacement was 11.8 ± 7.1 mm. The Euler angles in the Z-Y-X sequences were -1 ± 8, 1 ± 8, and − 8 ± 13 degrees. The correlation coefficient between the change in length and the displacement was − 0.724 (p < 0.001). The variables found to increase the risk of shortening and displacement were right-sided fracture (p = 0.037), male sex (p = 0.015), and multifragmentary type (p = 0.020). The variables found to increase the risk of rotation deformity were the number of rib fractures (p = 0.001) and scapula fracture (p = 0.025).

**Conclusions:**

There was a strong correlation between shortening and displacement. The magnitude of anterorotation around the X axis was greater than the magnitude of retraction around the Z axis and depression around the Y axis. The risk factors for shortening and displacement included right-sided fracture, male sex, and multifragmentary type. The risk factor for retraction around the Z axis was the number of rib fractures, and the risk factor for depression around the Y axis was scapula fracture. These results could be useful adjuncts in guiding minimally invasive surgical planning for diaphyseal clavicular fractures.

## Introduction

Minimally invasive surgery has been a widely performed treatment for vertebral fractures, pelvic fractures, and long bone fractures. However, a minimally invasive procedure for acute diaphyseal clavicular fractures has not been popular because of difficulties in performing a closed reduction of the fracture deformity of an injured S-shaped structure in a three-dimensional (3D) space [1, 2].

Specifically, deformities of the clavicle can be divided into translation deformities and rotation deformities. The translation deformity includes the change in clavicle length and the fracture displacement, and the rotation deformity includes three rotational degrees of freedom in the 3D space. Although the measurement methods of the change in length have been extensively studied, the measurement methods of the displacement and rotation deformity have rarely been discussed in previous studies [3–9].

Computed tomography (CT) with the 3D reconstruction technique has been widely used for injury assessment in recent years [9–11]. In comparison with plain radiography, CT could show the spatial features of the deformity more comprehensively. Therefore, we conducted this CT study to quantify the deformities of diaphyseal clavicular fractures. Moreover, we evaluated the correlation between the deformity parameters and identified the risk factors for deformities in the 3D space.

## Results

### Patient demographics and five deformity parameters

There were 72 men and 28 women, with an average age of 57.7 ± 14.6 years. There were 39 patients with right clavicle fractures and 61 patients with left clavicle fractures. The concomitant injuries included traumatic brain injury in 8 patients, ipsilateral scapula fracture in 20 patients, and ipsilateral rib fractures in 85 patients. There were 28 simple types, 43 wedge types, and 29 multifragmentary types.

Regarding the deformity parameters, the change in length of the endpoint line was − 5.3 ± 8.3 mm. There were 22 cases with positive values and 78 cases with negative values. The displacement was 11.8 ± 7.1 mm. The angle of rotation around the Z axis was − 1 ± 8 degrees, the rotation around the Y axis was 1 ± 8 degrees, and the rotation around the X axis was − 8 ± 13 degrees (Table 1).

**Table 1:** Patient demographics and deformity parameters

Patient demographics		Deformity parameters	
**Categorical**	**Number**	**Translation**	**Mean ± SD**
Sex		Change in length	− 5.3 ± 8.3 mm
Male	72	Displacement	11.8 ± 7.1 mm
Female	28	**Rotation**	**Mean ± SD**
Side		Rotation around Z	− 1 ± 8 degrees
Right	39	Rotation around Y	1 ± 8 degrees
Left	61	Rotation around X	− 8 ± 13 degrees
Brain injury			
Negative	92		
Positive	8		
Scapula			
Intact	80		
Fracture	20		
AO types			
Simple	28		
Wedge	43		
Multifragmentary	29		
**Continuous**	**Mean ± SD**		
Age	57.7 ± 14.6 years		
Rib fractures	4 ± 3 ribs		

*SD* standard deviation

### The correlation between the deformity parameters

**Fig. 1:**
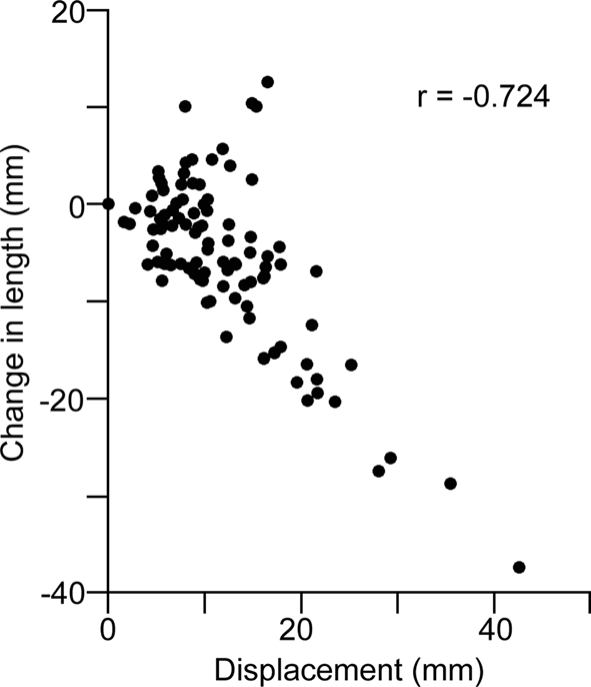
The correlation between the change in clavicle length and the displacement. The Pearson correlation coefficient between the change in length and the displacement was − 0.724 (P < 0.001)

The correlative analysis showed that the Pearson correlation coefficient between the change in length and the displacement was -0.724 (P < 0.001) (Fig. 1). Other combinations of the deformity parameters showed no statistically significant correlation (Table 2).

**Table 2:** Pearson correlation coefficient between radiographic parameters

	Length	Displacement	Rotation Z	Rotation Y	Rotation X
Length	1.000	− 0.724^†^	− 0.099	0.027	− 0.138
Displacement	–	1.000	0.145	0.025	− 0.015
Rotation Z	–	–	1.000	0.189	0.147
Rotation Y	–	–	–	1.000	− 0.070
Rotation X	–	–	–	–	1.000

^†^P < 0.001

### The risk factors for the deformity parameters

In univariable analysis, male patients (P = 0.004) and patients with multifragmentary types (P = 0.011) had more displacement. Patients with more rib fractures had more retraction around the Z axis (P = 0.001). Patients with concomitant scapula fractures (P = 0.004) had more depression around the Y axis. Although male patients and patients with left-sided fractures had more rotation around the Y axis, the difference was not statistically significant in multiple regression analysis (Table 3).

**Table 3:** The univariable analysis

	Change in length	Displacement	Rotation around Z	Rotation around Y	Rotation around X
Sex	P = 0.358	P = 0.004*	P = 0.810	P = 0.030*	P = 0.353
Male	− 5.8 ± 9.3 mm	13.0 ± 7.5 mm	− 1 ± 8 degrees	2 ± 8 degrees	− 9 ± 14 degrees
Female	− 4.1 ± 5.0 mm	8.6 ± 4.6 mm	− 1 ± 9 degrees	− 2 ± 8 degrees	− 6 ± 10 degrees
Side	P = 0.064	P = 0.594	P = 0.490	P = 0.020*	P = 0.666
Right	− 7.3 ± 9.0 mm	12.3 ± 8.1 mm	− 2 ± 7 degrees	− 1 ± 7 degrees	− 7 ± 14 degrees
Left	− 4.1 ± 7.7 mm	11.5 ± 6.4 mm	0 ± 8 degrees	2 ± 8 degrees	− 9 ± 13 degrees
Brain injury	P = 0.746	P = 0.533	P = 0.067	P = 0.830	P = 0.085
Negative	− 5.4 ± 8.6 mm	11.9 ± 7.1 mm	0 ± 8 degrees	1 ± 8 degrees	− 9 ± 14 degrees
Positive	− 4.4 ± 4.5 mm	10.3 ± 6.1 mm	− 6 ± 7 degrees	0 ± 6 degrees	0 ± 3 degrees
Scapula fracture	P = 0.402	P = 0.847	P = 0.938	P = 0.004*	P = 0.269
Negative	− 5.7 ± 8.2 mm	11.8 ± 6.8 mm	− 1 ± 7 degrees	0 ± 7 degrees	− 7 ± 13 degrees
Positive	− 3.9 ± 9.1 mm	11.5 ± 8.0 mm	− 1 ± 8 degrees	5 ± 11 degrees	− 11 ± 15 degrees
AO	P = 0.2094	P = 0.011*	P = 0.446	P = 0.813	P = 0.124
Simple	− 3.0 ± 6.5 mm	8.6 ± 5.9 mm	1 ± 8 degrees	2 ± 7 degrees	− 5 ± 12 degrees
Wedge	− 6.1 ± 7.9 mm	12.5 ± 6.1 mm	− 2 ± 8 degrees	1 ± 8 degrees	− 7 ± 13 degrees
Multifragmentary	− 6.5 ± 10.2 mm	13.8 ± 8.4 mm	− 1 ± 8 degrees	0 ± 8 degrees	− 12 ± 14 degrees
Age	P = 0.277	P = 0.255	P = 0.938	P = 0.679	P = 0.324
β	0.063	− 0.056	0.043	− 0.023	− 0.090
Rib fractures	P = 0.933	P = 0.776	P = 0.001*	P = 0.401	P = 0.636
β	0.026	− 0.074	− 0.917	− 0.244	− 0.231

*P < 0.05

In multiple regression analysis, patients with right-sided fractures had more shortening (P = 0.037). Male patients (P = 0.015) and patients with multifragmentary types (P = 0.020) had more displacement. Patients with more rib fractures had more retraction around the Z axis (P = 0.001). Patients with concomitant scapula fractures (P = 0.025) had more depression around the Y axis (Table 4).

**Table 4:** Multiple regression analysis

	Change in length		Displacement		Rotation around Z		Rotation around Y		Rotation around X	
	Stand. β	P	Stand. β	P	Stand. β	P	Stand. β	P	Stand. β	P
Sex	0.104	0.345	− 0.262	0.015*	0	1.000	− 0.131	0.223	0.053	0.629
Side	0.218	0.037*	− 0.120	0.228	0.050	0.612	0.178	0.077	− 0.015	0.886
Brain injury	0.038	0.705	− 0.053	0.587	− 0.189	0.055	0.029	0.767	0.171	0.094
Scapula fracture	0.087	0.432	− 0.068	0.522	0.011	0.915	0.243	0.025*	− 0.103	0.352
AO1	− 0.188	0.133	0.234	0.052	− 0.201	0.094	− 0.123	0.308	− 0.035	0.778
AO2	− 0.167	0.180	0.281	0.020*	− 0.114	0.339	− 0.094	0.433	− 0.243	0.051
Age	0.080	0.443	− 0.040	0.688	0.045	0.653	− 0.024	0.812	− 0.113	0.278
Rib fractures	− 0.014	0.889	0.016	0.872	− 0.327	0.001*	− 0.075	0.450	− 0.036	0.724
*Adjusted R*^*2*^	0.018		0.089		0.090		0.075		0.027	

*P < 0.05; Stand., standardized

There were 72 patients with the injured arm down and 28 patients with the injured arm up. The patients with the injured arm up had more retraction around the Z axis (P = 0.004) and less anterorotation around the X axis (P = 0.004) (Table 5).

**Table 5:** The influence of arm positions on fracture deformities

	Arm down (n = 72)	Arm up (n = 28)	P value
Length	− 5.8 ± 9.4 mm	− 4.2 ± 4.8 mm	0.381
Displacement	12.3 ± 7.7 mm	10.5 ± 5.0 mm	0.244
Rotation Z	1 ± 8 degrees	− 4 ± 8 degrees	0.004*
Rotation Y	1 ± 9 degrees	0 ± 6 degrees	0.326
Rotation X	− 10 ± 14 degrees	− 2 ± 10 degrees	0.004*

*P < 0.05

## Discussion

The translation deformity of diaphyseal clavicular fractures includes the change in clavicle length and the fracture displacement. The rotation deformity includes the rotation around the X, Y, Z axes. The current study showed that there was a significant correlation between the magnitude of the change in length and the displacement. Patient characteristics, such as male sex, right-sided fracture, and multifragmentary type, were risk factors for translation deformities of diaphyseal clavicular fractures. On the other hand, concomitant injuries to adjacent bony structures, such as scapula fractures or more rib fractures, lead to rotation deformities.

Regarding the correlation between the deformity parameters, the current study showed that there was a strong correlation between the magnitude of the change in length and the displacement. This finding was consistent with the surgical observation. The more displacement the fracture has, the more concerned we should be about shortening. This result also indicated that defining the surgical indication of shortening and displacement at the same level of 20 mm is reasonable in clinical practice [12, 13]. However, other combinations of the deformity parameters showed no statistically significant correlation in the current study.

The risk factors for translation deformities can be analyzed from several aspects. First, the current study showed that the average change in length was − 5.3 ± 8.3 mm, and the average displacement was 11.88 ± 7.1 mm. Even though more than 80% of patients were diagnosed with polytrauma injuries, the average shortening and displacement were both less than 20-mm surgical indication [12, 13]. The multiple regression analysis also showed that concomitant injuries were not risk factors for translation deformities. These findings indicated that although polytrauma patients were more likely to have concomitant injuries in close proximity to the clavicle, such as rib fractures or a scapula fracture, these injuries would not increase the surgical necessity of clavicle fracture at initial presentation. Second, there were 22 cases with lengthening of the endpoint line in the current study, which might be attributed to the gravity-assisted reduction of acute fractures in a supine position during CT examination [3–11]. From the clinical point of view, for patients with more shortening of acute clavicular fracture, the supine position might assist us in restoring the clavicle length. Third, although the multiple regression analysis showed that right-sided fracture was a risk factor for shortening, this result might vary in different countries because the trend of traffic-related injuries could be influenced by different driving rules.

Rotation deformities include three rotational degrees of freedom in 3D space. The current study revealed that the magnitude of anterorotation of acute diaphyseal clavicular fracture was larger than the magnitude of retraction or the magnitude of depression. Because rotation deformities are difficult to evaluate during minimally invasive surgery, these results could be useful adjuncts in guiding reduction techniques. In addition, due to a lack of appropriate structural balance, more rib fractures resulted in an increasing retraction angle, and scapular fractures resulted in an increasing depression angle. From the clinical point of view, when surgery is necessary for a patient with multiple rib fractures, a sandbag behind the shoulder could be considered to neutralize the retraction force, which could elevate the distal main fragment off the operation table to assist reduction. For a patient with ipsilateral scapula fracture, the supine position might be a better option than the beach chair position to decrease the gravity-induced depression force.

Based on recommendation, the arms should be in the arms-up position during scanning to obtain clearer images. However, patients with polytrauma or shoulder girdle injuries could not raise their injured arm. In the current study, the influence of arm positions on fracture deformities was analyzed. The patients with the injured arm up had more retraction around the Z axis and less anterorotation around the X axis. Although the patients with their injured arm up had less depression around the Y axis, the difference was not statistically significant. From the clinical point of view, a depression deformity cannot be sufficiently reduced by simply raising the humerus. An upward force may need to be applied additionally.

The contralateral uninjured clavicle was used as the template in the current study. Although between-side differences exist in the length and rotation alignment of the native human clavicle, a previous study showed that the differences were only 3 mm and 3 degrees [9]. Using the contralateral uninjured clavicle as the template is still the most appropriate strategy for virtual reduction of the injured fragments.

There were some limitations of this study. First, because of the retrospective nature of this study, the quality of the CT images was not excellent. Second, the rotation deformity was defined as the rotation of the distal main fragment relative to the proximal main fragment. Nevertheless, the relative positions of the proximal main fragment in the coordinate frame of the whole body were not calculated. Third, because patients underwent CT examination in a supine position at initial presentation, the fracture deformities might be different from the deformities of long-term follow-up [9, 14]. Fourth, most patients in the current study had concomitant injuries. There might be morphological differences between patients with concomitant injuries and patients with isolated fractures.

## Conclusion

There was a significant correlation between the shortening and displacement. Patient characteristics, such as male sex, right-sided fracture, and multifragmentary type, were risk factors for translation deformities. Concomitant injuries, such as rib fractures and ipsilateral scapula fractures, were risk factors for rotation deformities. Patients with more rib fractures had more retraction deformities, and patients with scapular fractures had more depression deformities.

## Materials and methods

This was a retrospective study. Institutional review board approval was obtained.

### Patient demographics

At a level one trauma center, we searched the CT images of patients who sustained an acute diaphyseal clavicular fracture between 2011 and 2018. After patients with preexisting clavicle injuries or patients with incomplete imaging data were excluded, 100 patients with an acute unilateral clavicle fracture were included in this study. Patient age, sex, injury side, traumatic brain injury, number of rib fractures, scapular integrity, OTA/AO classification of the fracture pattern, and the arm positions during scanning were recorded [15].

### Preparation of the markers

**Fig. 2:**
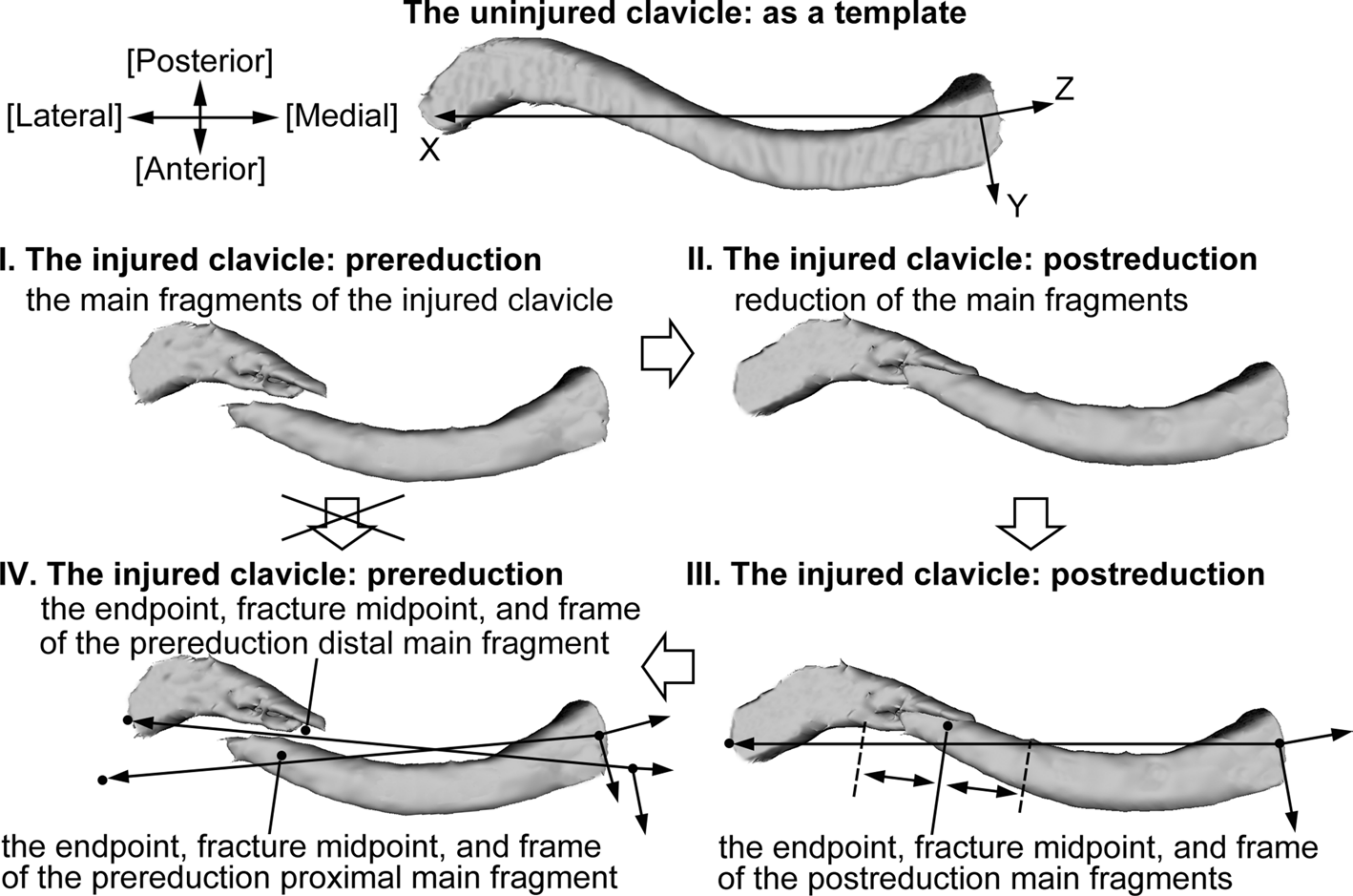
Preparation of the endpoint, fracture midpoint, and frame. Because it was not credible to directly place these markers on the injured and deformed fragments, we used postreduction images to make the measurement process more accurate. The uninjured clavicle was used as a template. The 3D images of the main fragments of the injured clavicle were identified and reduced to the template. After identifying the endpoints, fracture midpoint, and frame in the postreduction status, we transformed the markers back to the prereduction position to obtain the initial coordinates

Every uninjured clavicle was flipped to the right side to establish the template and the local coordinate frame in 3D Slicer [16]. The X axis was defined by the endpoint line containing the two articular centers. The Y axis was defined by the widest diagonal of the projection of the centerline on the plane perpendicular to the endpoint line. The Z axis was perpendicular to the X and Y axes. The X, Y, Z axes were the left-to-right, posterior-to-anterior direction, and inferior-to-superior direction, respectively (Fig. 2).

Three kinds of markers were used to describe the 3D deformities of an injured clavicle: the endpoints were used to assess the change in clavicle length, the fracture midpoints were used to assess the fracture displacement, and the coordinate frames were used to assess the rotation deformities of the main fragments. Because it was not credible to directly place these markers on the injured and deformed fragments, we used postreduction images to make the measurement process more accurate. The preparation process could be divided into four steps. First, the injured clavicle was flipped to the right side to create the 3D image. The proximal main fragment and the distal main fragment were identified. Second, the main fragments of the injured clavicle were reduced virtually within the uninjured clavicle, which was used as a template. Third, we identified the endpoints, fracture midpoint, and frame of the main fragments in the postreduction status. The fracture midpoint was defined as the midpoint between the most proximal point on the fracture edge of the proximal main fragment and the most distal point on the fracture edge of the distal main fragment. Fourth, the position of the endpoints, fracture midpoint, and frame of the main fragments in the postreduction status was transformed back to the corresponding prereduction position (Fig. 2).

### The five deformity parameters

**Fig. 3:**
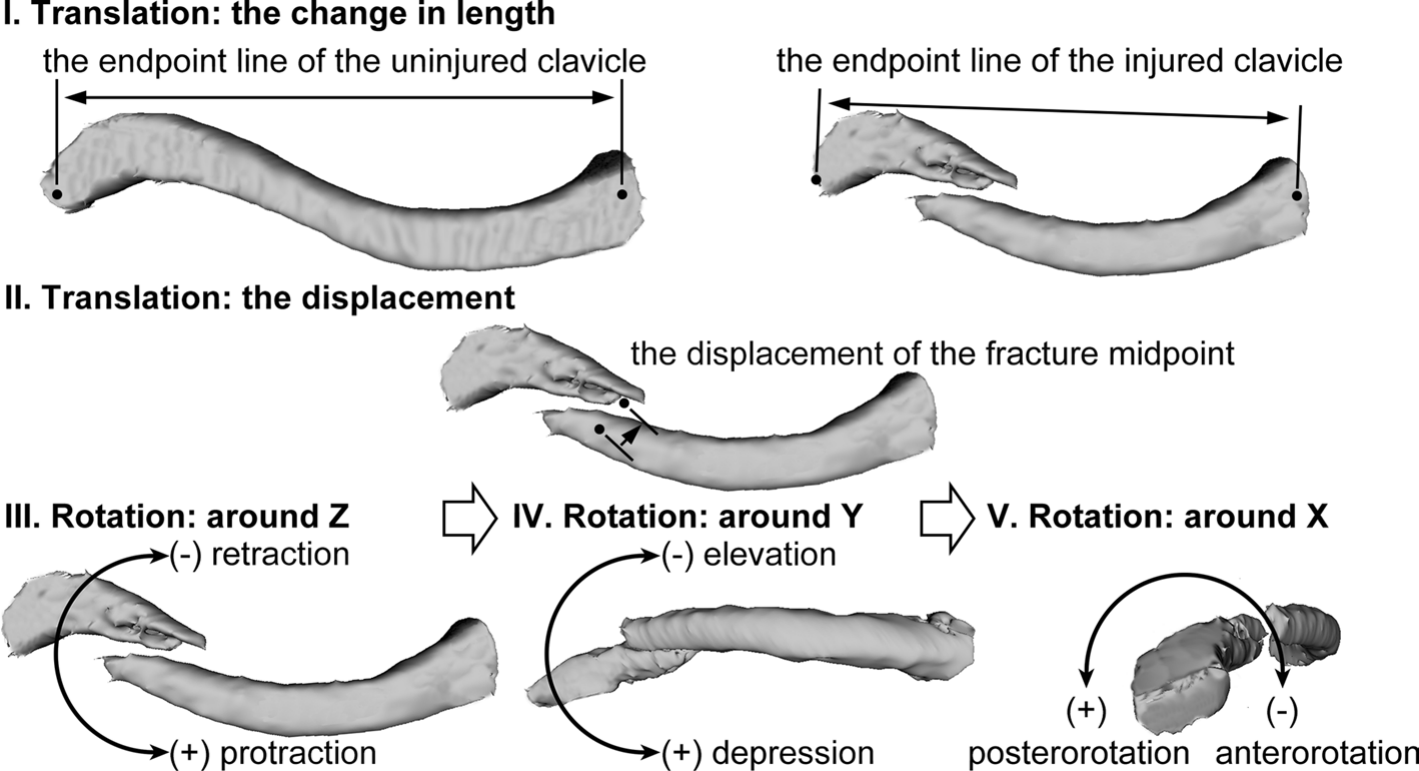
The five deformity parameters. The change in clavicle length was defined as the change in length of the endpoint line. The fracture displacement was defined as the distance between the fracture midpoint of the prereduction proximal main fragment and the fracture midpoint of the prereduction distal main fragment. The rotation deformities were defined as the Euler angles in the Z-Y-X sequence

The five deformity parameters included the change in clavicle length, fracture displacement, and rotation around the X, Y, Z axes. The change in length was the difference in length between the endpoint line of the uninjured clavicle and the endpoint line of the injured clavicle. Negative values represent shortening, and positive values represent lengthening. The fracture displacement was defined as the distance between the fracture midpoint of the prereduction proximal main fragment and the fracture midpoint of the prereduction distal main fragment. Rotation deformity was defined as the rotation of the frame of the prereduction distal main fragment relative to the frame of the prereduction proximal main fragment in 3D space. The Euler angles in the Z-Y-X sequence were used in the current study (Fig. 3).

### Statistical analysis

The five deformity parameters are represented as the mean and standard deviation (SD). The relations between the five deformity parameters were analyzed using the Pearson correlation coefficient. In regression analysis, sex, injured side, traumatic brain injury, scapula fracture, OTA/AO classification, patient age, and the number of rib fractures were regarded as independent variables. The five deformity parameters were regarded as dependent variables. In univariable analysis, Student’s t test was used for dichotomous variables, ANOVA was used for trichotomous variables, and simple linear regression was used for continuous variables. In multiple regression analysis, simultaneous regression models were developed for each of the five dependent variables to obtain the standardized regression coefficient. The arm position on the injured side was classified as the injured arm down or the injured arm up. Student’s t test was used to investigate the influence of arm positions on fracture deformities. All p values less than 0.05 were considered significant.

## Data Availability

The data are available from the author upon reasonable request.

## Abbreviations

3D: Three-dimensional
CT: Computed tomography
SD: Standard deviation
Stand.: Standardized
